# Investigation of antioxidant and anticancer activities of unsaturated oligo-galacturonic acids produced by pectinase of *Streptomyces hydrogenans* YAM1

**DOI:** 10.1038/s41598-021-87804-9

**Published:** 2021-04-19

**Authors:** Afrouzossadat Hosseini Abari, Hamed Amini Rourani, Seyed Mahdi Ghasemi, Hyun Kim, Yun-Gon Kim

**Affiliations:** 1 Department of Cell and Molecular Biology & Microbiology, Faculty of Biological Sciences and Technology, University of Isfahan, Isfahan, Iran; 2 Environmental Research Institute, University of Isfahan, Isfahan, Iran; 3 Department of Biotechnology, Faculty of Biological Sciences and Technology, Shahid Ashrafi Esfahani University, Isfahan, Iran; 4 School of Chemical and Biological Engineering, Seoul National University, Gwanak-ro 1, Gwanak-gu, Seoul, 08826 Republic of Korea; 5 Department of Chemical Engineering, Soongsil University, Seoul, 06978 Republic of Korea

**Keywords:** Applied microbiology, Environmental microbiology

## Abstract

Pectin, a diverse carbohydrate polymer in plants consists of a core of α-1,4-linked D-galacturonic acid units, includes a vast portion of fruit and agricultural wastes. Using the wastes to produce beneficial compounds is a new approach to control the negative environmental impacts of the accumulated wastes. In the present study, we report a pectinase producing bacterium *Streptomyces hydrogenans* YAM1 and evaluate antioxidative and anticancer effects of the oligosaccharides obtained from pectin degradation. The production of oligosaccharides due to pectinase activity was detected by thin layer chromatography (TLC) and matrix-assisted laser desorption ionization time-of-flight mass spectrometry (MALDI-TOF MS). Our results revealed that *S. hydrogenans* YAM1 can degrade pectin to unsaturated pectic oligo-galacturonic acids (POS) with approximately 93% radical scavenging activity in 20 mg/mL which it is more than 50% of the same concentration of pectin. Flow cytometric analysis revealed that MCF-7 cells viability decreased more than 32 and 92% following treatment with 6 and 20 mg/mL POS after 24 h, respectively. It is suggested that pectin degradation by *S. hydrogenans* YAM1 is not only a new approach to produce highly active compounds from fruit wastes, but also is an effective method to remove fibrous pollutants from different environments.

## Introduction

According to increasing the world population, agricultural and food wastes create environmental stress with a lost US$1 trillion in a year^1^. Global scenario to solve this problem is waste valorization and the utilization of agri-food waste or by-products to produce valuable products applicable for food, cosmetics or pharmaceutical industries. In this way, most of the wastes have been utilized as organic fertilizers or as a source of fuel. In the concept of biorefinery, the wastes have been used to produce bioethanol, enzymes, and some other beneficial compounds to control the negative environmental impacts of the accumulated wastes^2^. A new approach to use the remained fruit fibers is microbial hydrolyzing of them as natural polymers to synthesize beneficial oligomers^3^.

Most of the fruit wastes arise after pressing the juice and the remained pomace and non-edible parts of fruits. The waste is contained higher amounts of bioactive compounds such as oligo- saccharides, polyphenols, carotenoids, dietary fibers, enzymes, vitamins, and oils^4,5^. Nowadays, scientists encourage people to add more fruits and juices in their daily diet. About half of the used fruits annually remain as wastes such as fibers (cellulose, lignin, starch, pectin, and hemicelluloses). Apple is one of the most consuming fruit due to wide benefits for human health which is consisting of a significant part of pectin. A significant portion of the world’s pectin production is extracted from apple pomace^6^.

Pectin, a polymeric diver’s matrix in the plant cell wall, consists of a core of *α*-1,4-linked D-galacturonic acid units with a various number of natural sugars such as rhamnose, arabinose, galactose, and less amount of fucose, aceric acid, apiose, xylose and glucuronic acid^7^.

Previous studies have shown that carboxyl, hydroxyl and methoxy groups can enhance the antioxidant activity of lignin and other phenolic compounds^8,9^. Therefore, the presence of various carbohydrates especially galacturonic acids with several electron donating groups can increase antioxidant activity of pectin and its derivatives. According to the therapeutic effects and low toxicity of pectin and pectic oligo-saccharides (POS), they have wide applications in the food and pharmaceutical industries^10,11^. Pectin has been recognized as a food supplement to reduce the risk of colon cancer because of the anti-inflammation effects. Pectin and POS rich diets may block caspase-3 abilities to the promotion of tumor cell migration and they also decrease anti-apoptotic Bcl2 protein expression at the same time^12,13^. Anticancer effects of commercialized products of POS obtained from treated pectin such as GCS-100 and PectaSol, have been studied in many cancers such as breast, head, and prostate cancer^14,15^. The results demonstrated that POS have anticancer effects on MDA-MB-231 (Human breast cancer cells), and human prostatic carcinoma cell line (LNCaP and LNCaP C4-2 cells) without any toxic effects on non-cancer cells^16^. This is the biggest competency of POS in comparison with other non-natural anti-cancer drugs.

Pectic oligo-saccharides have been obtained from various methods such as alkali or acidic pH, heat, and enzymatic treatment^17^. Enzymatic treatment of pectin has been widely performed by pectinolytic enzymes obtained from fungi and bacteria which are industrially used in fruit juice clarification and plant food quality improvement. The enzymes are divided into three groups: Hydrolases, consisting of polygalacturonase and pectin hydrolase which have been reported in *Aspergillus niger* and *Erwinia* sp., catalyze pectate and pectin to mono-, di-, oligo- galacturonates or mono-, oligo- methyl galacturonates^18^. Lyases, pectin and pectate lyases, which catalyze pectin to unsaturated forms of galacturonate or methyl galacturonate are widely distributed in *Aureobasidium pullulans*, *Pichia pinus*, *Bacillus licheniformis* and *Bacteroides*^19–22^. Pectin methyl esterase (PME), which randomly cleaves the methyl ester group of galacturonate, and is commercially used in fruit juice clarification, has been reported in a broad range of fungi and plant pathogenic microorganisms such as *Rhodotorula* sp., *Saccharomyces cerevisiae*, *Erwinia chrysanthemi* B341 and *Pseudomonas solanacearum*^15,23–25^.

Thermal and pH stable activities of pectinases have been reported in *Streptomyces*, the high noticeable genus of *Actinobacteria*^26,27^. Consequently, it is interesting to explore new streptomycetes with improved enzymatic potential.

*Streptomyces hydrogenans*, a member of *Actinobacteria* from the family of *Streptomycetaceae*, is applicable for biocontrol of insect pests due to its insecticidal activities. It can also produce actiomycine-D which is an antibacterial and antifungal agent^28,29^. This study was aimed to investigate pectin degradation by pectinase of *S. hydrogenans* YAM1. The presence of galacturonic acids with several methoxy, carboxyl and hydroxyl groups is the probable reason of antioxidant activity of pectin and its derivatives. The main objective of this research was to monitor the bioactive properties of the products obtained from pectin degradation.

## Results and discussion:

### Identification of the isolated strain and monitoring the pectinase activity

**Figure 1:**
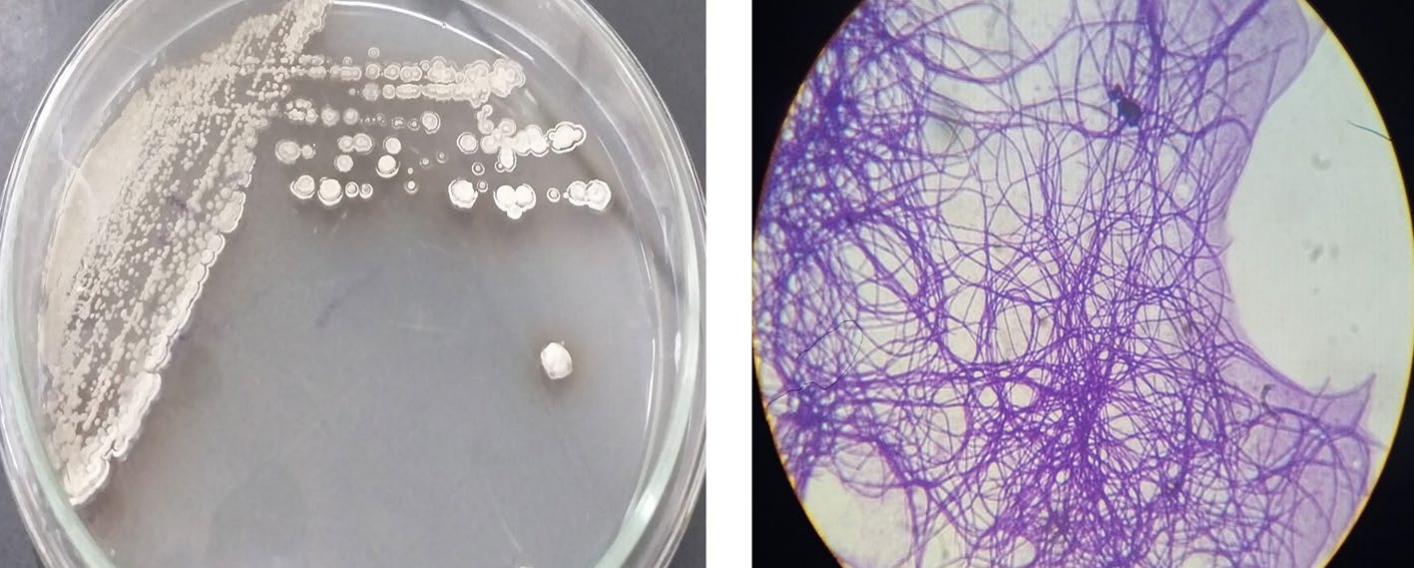
*Streptomyces hydrogenans* YAM1 colonies on pectin agar and the microscopic observation of the cells.

**Figure 2:**
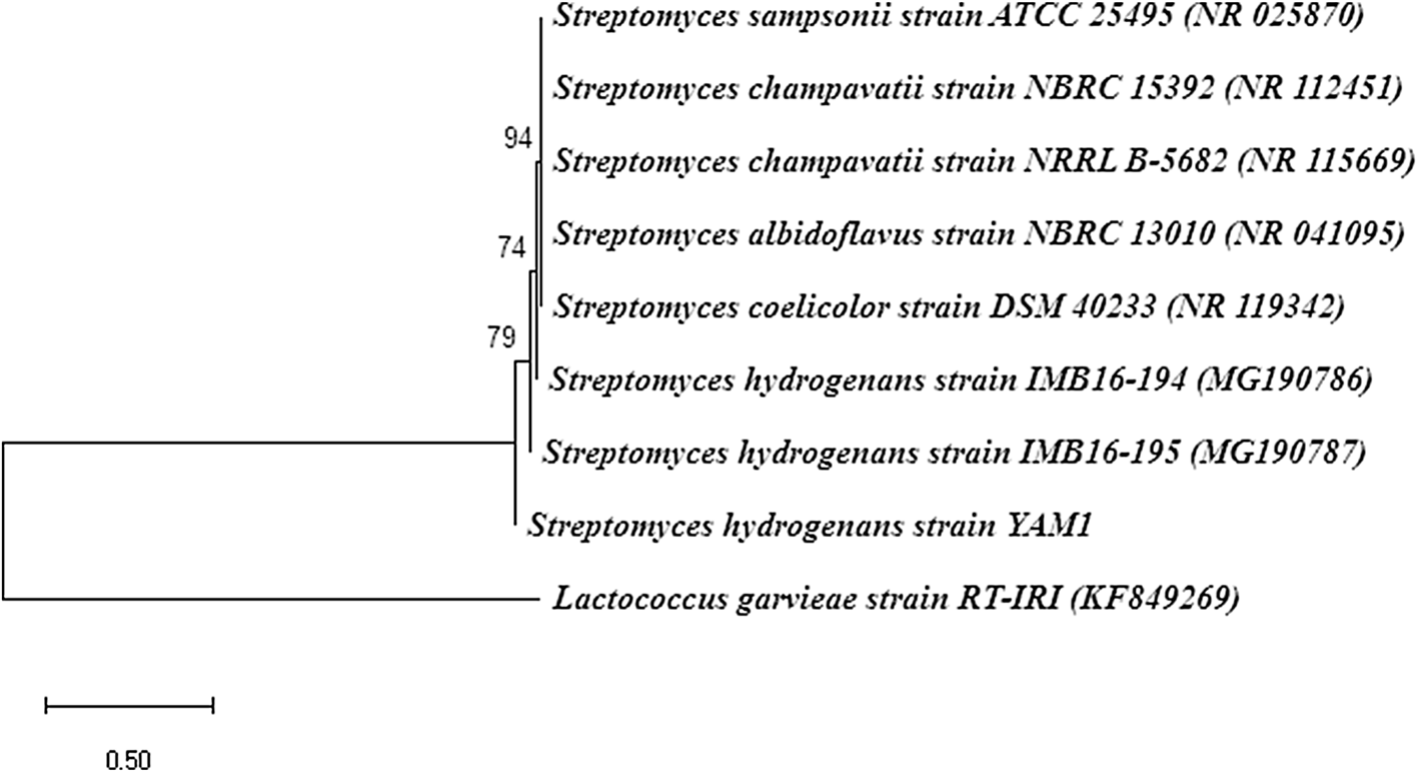
Phylogenetic relationships of *Streptomyces hydrogenans* strain YAM1. The phylogenetic tree was constructed based on 16S rDNA sequences, using the neighbor-joining method with 2000 bootstrap replications.

The isolated strain was Gram-positive, aerobic, non-acid-fast with the optimal growth at 30˚C and pH 6.8 to 7. The colonies were white with the powdery aerial mycelium (Fig. 1). The phylogenetic analysis revealed that the isolated strain belongs to the phylum *Actinobacteria*, the family *Streptomycetaceae,* and the genus *Streptomyces*. Based on the BLAST analysis, it was submitted as *Streptomyces hydrogenans* YAM1 (MN255488) to NCBI. As shown in Fig. 2, phylogenetic analysis using the neighbor-joining method confirmed that the isolated strain is related to *S. hydrogenans*.

*S. hydrogenans* is recognized as an antifungal and insecticidal bacterium with significant potential in biocontrol studies^28,29^. Metabolites produced by *S. hydrogenans* also enhance growth and yield production with an effective role in biotic stress mitigation in plants^30^. The metabolites eradicate nematodes that infect plants and are a safe alternation to chemical pesticides in crop protection. Although *S. hydrogenans* is recognized as an effective bacterium in biocontrol, the study on its other potentials is still incomplete.

**Figure 3:**
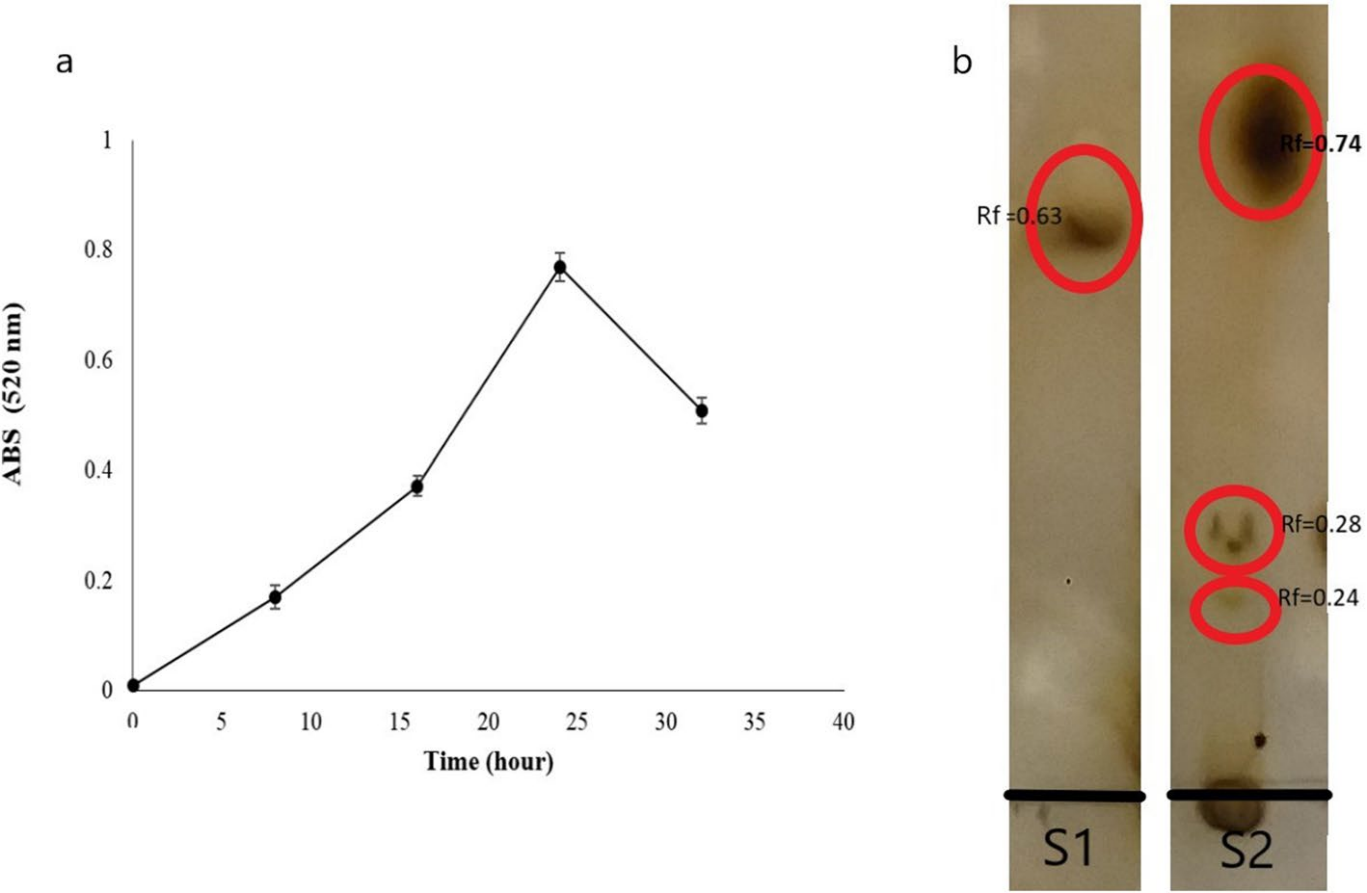
Monitoring of the pectinase activity in *Streptomyces hydrogenans* YAM1. (**a**) Releasing of sugars from the pectin degradation during the time. (**b**) TLC pattern of the obtained products, S1: standard solution of mono- galacturonic acid, S2: the pectic oligosaccharides from pectin degradation.

The amount of released sugars from pectin degradation by pectinase activity of *S. hydrogenans* YAM1 was measured by DNS reagent. Considering the goals of the study, the experiment was performed to determine the time of the highest amount of sugar release during pectin degradation by *S. hydrogenans* YAM1. The results demonstrated the maximum concentration of the released sugars in 24 h incubation at 30 ºC, 180 rpm (Fig. 3a). Henceforth, TLC was used for the investigation of the obtained products. The patterns revealed that the strain was able to degrade pectin to oligo-galacturonic acids (Fig. 3b). The products of the enzymatic degradation then were lyophilized for further analyses.

### MALDI-TOF/MS for analysis of the obtained oligomers

**Figure 4:**
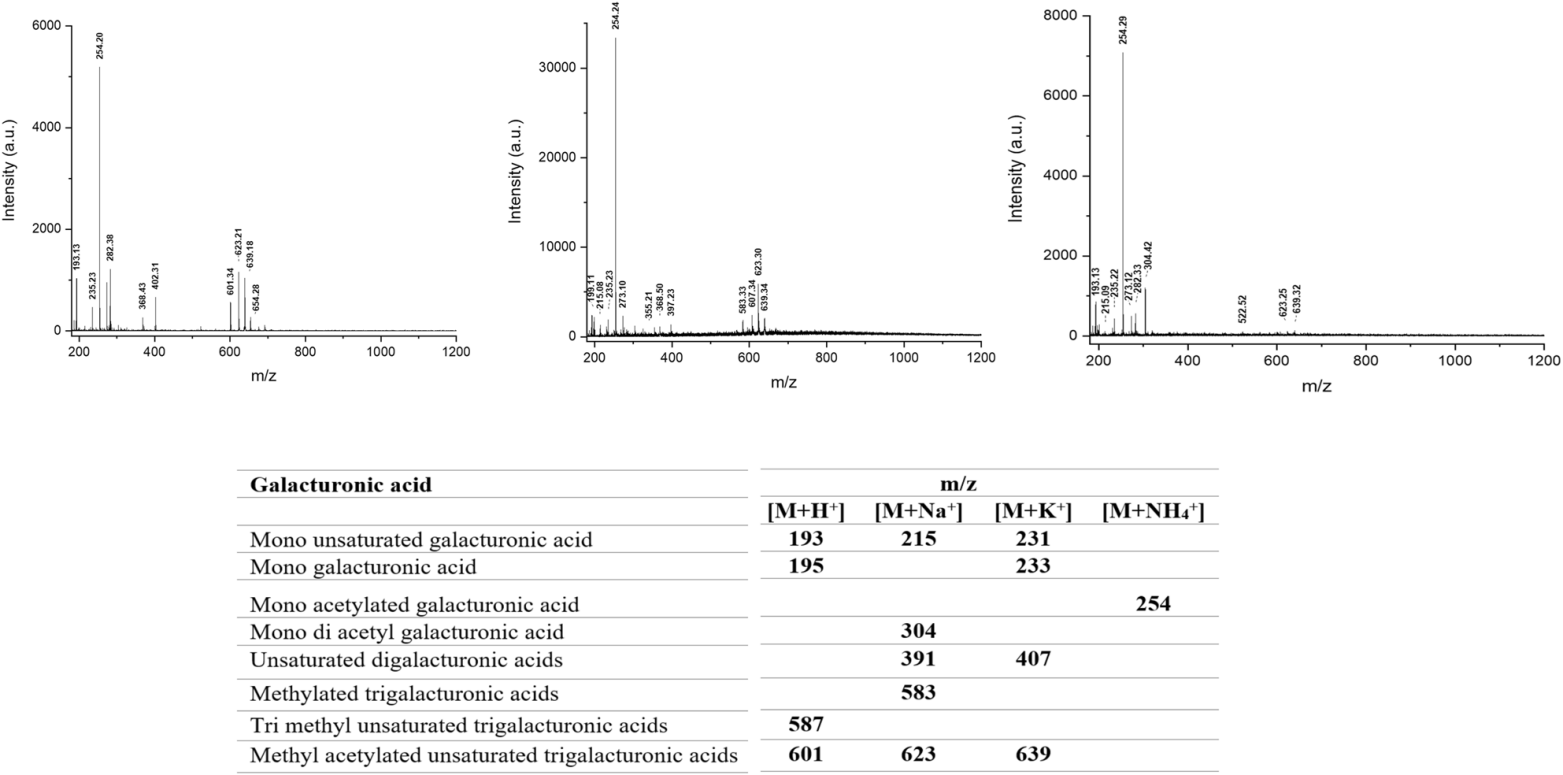
MALDI TOF/MS spectroscopy of pectic oligo-saccharides obtained from pctin degradation by *Streptomyces hydrogenans* YAM1.

Pectic oligo-galacturonates produced from pectin degradation by *S. hydrogenans* were verified by MALDI-TOF/MS in triplicate analyses (Fig. 4). The MALDI-TOF mass spectrums of this fraction showed the presence of mono galacturonic acid and unsaturated forms of mono, di, and tri galacturonic acids in the sample. Pectin lyase catalyzes the cleavage of pectin, producing unsaturated oligogalacturonates^31^. According to the type of the products obtained from pectinase activity of *S. hydrogenans*, it is supposed that the pectinolytic activity observed in this study is due to pectin lyase.

Pectinases are more reported in fungi such as *Aspergillus, Alternaria and Penicillium* than the bacterial species. Polygalacturonases have been reported in various types of bacteria such as *Acinetobacter guillouiae*, *Kosakonia sacchari, Bacillus vallismortis*^32^. Pectate and pectin lyases have been found in actinomycetes*, Pseudomonas and Bacillus* species, and some plant pathogenic bacteria such as *Xanthomonas campestris* pv. *campestris, Dickeya dadantii* and *Pectobacterium atrosepticum*^21,33^*.* Pectate lyase is also observed in a human gut bacterium* Bacteroides thetaiotamicron* VPI-5482^34^.

### Detection of antioxidative effects

**Figure 5:**
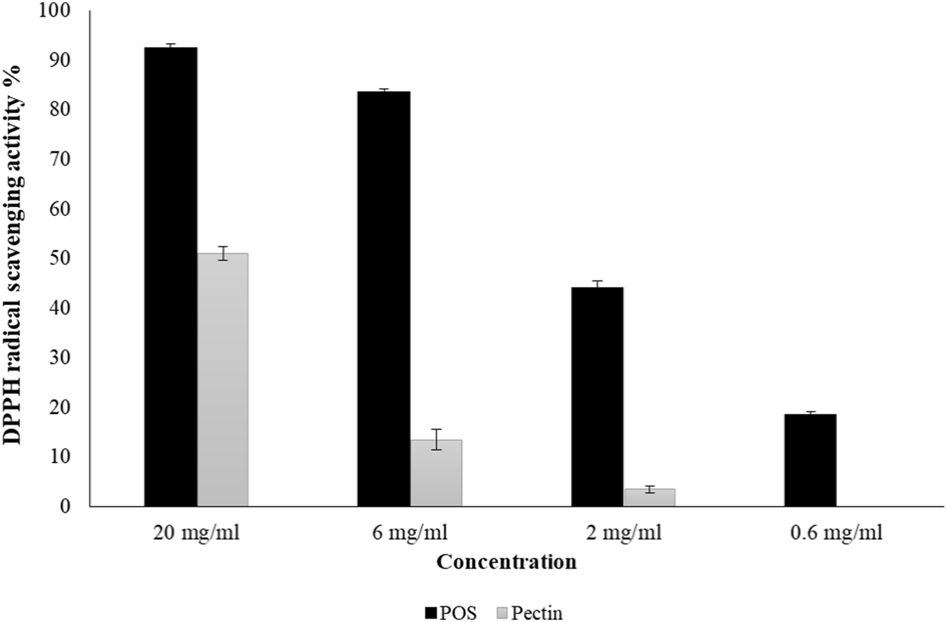
DPPH radical scavenging activity of pectin and pectic oligo-saccharides.

Antioxidative effects of POS (from 0.6 to 20 mg/mL) and the same concentration of pectin were tested by DPPH. The results revealed that radical scavenging activity was increased as the concentration of POS and pectin raised. As shown in Fig. 5, 0.6 to 20 mg/mL of POS showed significant radical scavenging activity from 19 to 93%. The POS exhibited more than 50% DPPH radical scavenging activity compared to the same concentration of pectin. The results also demonstrated that the POS approximately have 19% DPPH scavenging activity in the concentration 0.6 mg/mL. Whereas, pectin did not show any antioxidant activity in the same concentration. Antioxidant activity of pectin and pectin derivatives have been reported in previous studies^35–38^. Antioxidants can prevent the oxidative stress caused by environmental pollution, exposure to chemical pesticides or drugs, radiation, consumption of processed foods and certain additives. The DPPH scavenging activity of pectin is closely related to the presence of hydroxyl groups. Chen et al. demonstrated antioxidant activity in a concentration-dependent manner of POS obtained from microwave-assisted extraction of tangerines peel^35^. Ogutu and Mu reported that the degradation of potato pectin by ultrasonic significantly increased its antioxidant activity^36^. In all the similar experiments, antioxidant activities of POS were dose-dependent. In this study, 20 mg/mL unsaturated pectic oligosaccharides obtained from pectin degradation by *S. hydrogenans* YAM1 showed more than 90% DPPH scavenging activity. The results also represented an increase in the antioxidant property of pectin after digestion by pectinase which had been confirmed in previous researches^37,38^. Furthermore, previous studies revealed that electron donating groups such as carboxyl, hydroxyl and methoxy groups can enhance the antioxidant activity of phenolic compounds^8,9^. Lignin antioxidant properties are also related to methoxy groups and phenolic hydroxyl. Although the radical scavenging activity of hydroxyl groups in polysaccharides was minor because of lacking phenolic structure, many other factors such as the presence of galacturonic acid, and other chemical components in polysaccharides, were assumed to play an important role in their antioxidant activities^39^.

On the other hand, there is a correlation between molecular weight and radical scavenging activity of polysaccharides. Polysaccharides with low molecular weights could have more available reductive hydroxyl group terminals to eliminate the free radicals. Degradation of a polymer is accompanied with a decrease in molecular weight, hence improving the antioxidant potentials of polysaccharides^17^. Thus, degradation of pectin to oligosaccharides with low molecular weights and the presence of various carbohydrates especially galacturonic acids with several electron donating groups is the main reason of enhanced antioxidant activity of POS.

There are different methods for degradation of pectin to produce subunits with lower molecular weights. Among them, pH treatment, heat treatment and enzymatic treatment of pectin are the most used methods^39^. Enzymatic hydrolysis of pectin has advantages over the other hydrolysis methods as enzymes target specific linkages of the pectin molecules, while the other methods showed less specificity. This is especially important when partial hydrolysis is required to obtain specific fragments. For this purpose, pectinolytic enzymes that cleave specific linkages are required to produce desired oligosaccharides, without further hydrolysis. Therefore, different degradation methods could produce POS with different structures and molecular weights which can affect antioxidant activity^40^.

### Effect of oligo-galacturonic acids on MCF-7 breast cancer cell viability

**Figure 6:**
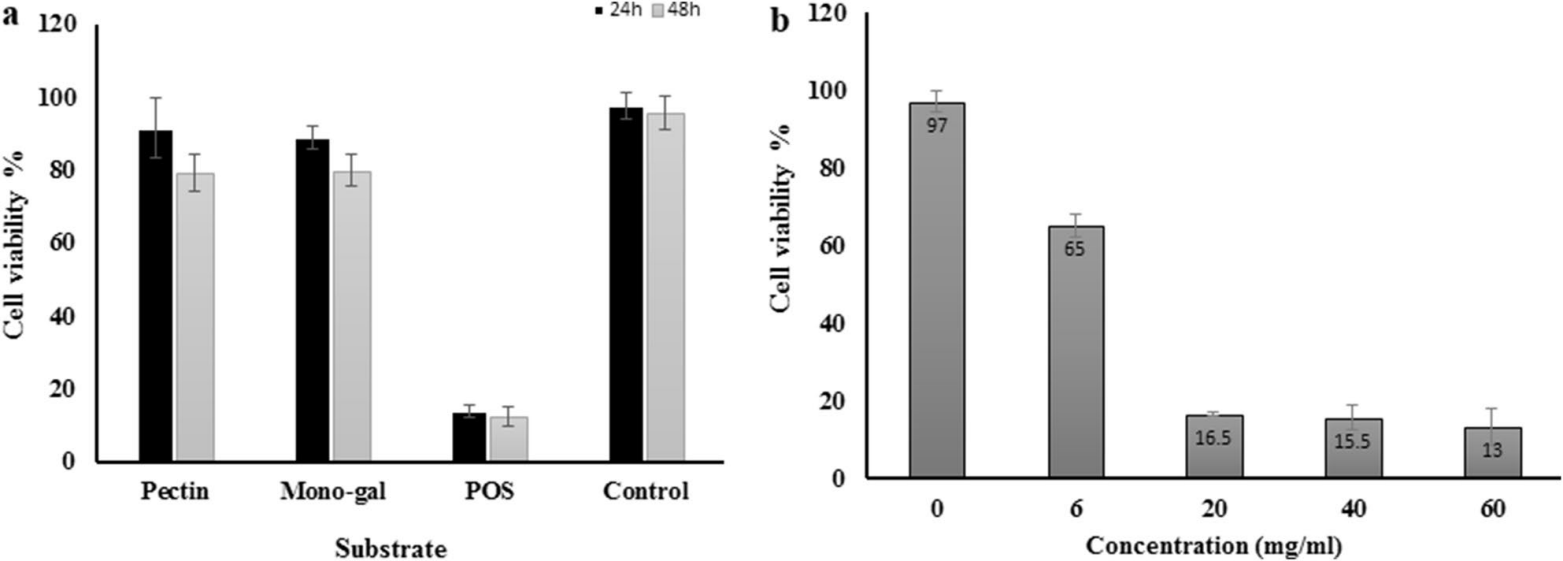
MTT analysis of MCF-7 cell viability after 24 h. (**a**) represented significant differences (*P* < 0.05) of cell viability percentage among 20 mg/mL of pectin, Mono-galacturonic acid, pectic oligo- saccharide. (**b**) cell viability percentage of MCF-7 treated cells by 0 to 60 mg/mL POS.

As shown in Fig. 6a, POS showed significantly more anticancer activity against MCF-7 cells than the same concentration of pectin and mono-galacturonic acids. The viability results indicated the incredible improvement of pectin anticancer effects after hydrolysis and formation of POS. Evaluation of cell viability by MTT assay demonstrated a minimum of 35% and a maximum 87% viability reduction in 6 and 60 mg/mL of POS after 24 h incubation, respectively (Fig. 6b). As shown, no significant differences in cell viability were observed among 20, 40, and 60 mg/mL concentrations.

**Figure 7:**
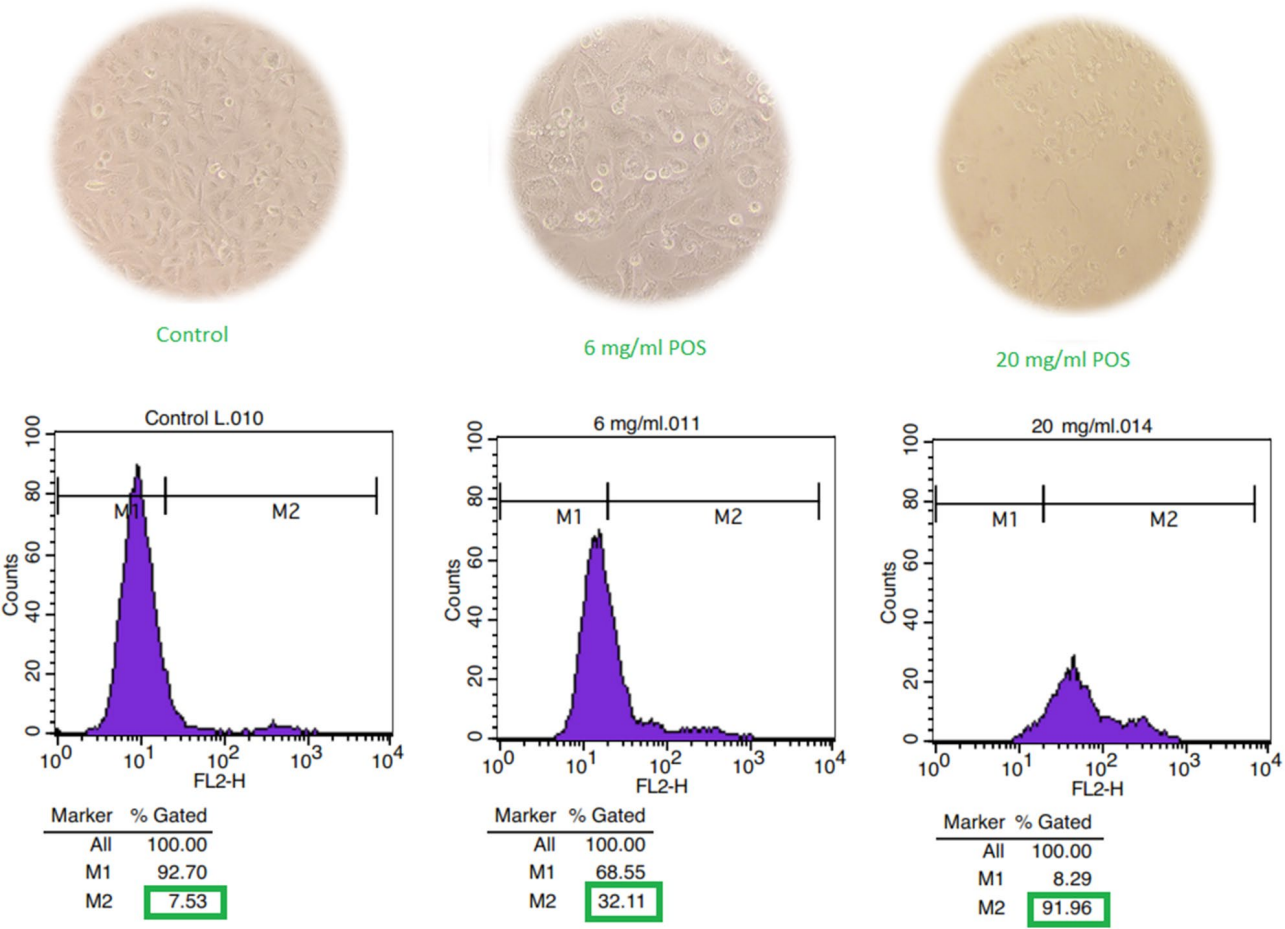
Morphological changes and flow cytometery histograms of POS treated MCF-7 after 24 h. M1 zone represents the position of living cells that were not stained by PI, and the M2 zone shows the spreading of dead cells. The cell viability is 92.70%, 68.55%, and 8.29% in non-treated control, 6 and 20 mg/mL POS treated cells, respectively.

The morphological changes of the treated cells and flow cytometric analysis is displayed in Fig. 7. As shown, induction of death was monitored in 6 and 20 mg/mL POS treated MCF-7 cells. Flow cytometric analysis also revealed that MCF-7 cell viability decreased 32 and 92% following treatment with 6 and 20 mg/mL POS after 24 h, respectively. The location of living cells which were not stained by PI is represented as M1 zone and the distribution of dead cells was demonstrated as M2 zone. As shown, the cell viability of 92, 68, and 8% was observed in non-treated control, 6 and 20 mg/mL POS treated cells, respectively.

Due to the disadvantages of chemotherapy in cancer treatment such as the drug resistance of tumor cells, and serious side effects on healthy cells, using natural anticancer products has recently gathered much attention. Many studies have been demonstrated the induction of apoptosis and the decrease of cell proliferation by pectin or pectin derivatives^41^. Kapoor and Dharmesh evaluated the anticancer effect of POS isolated from Sour Raw Tomato (SrTPO) on the AGS gastric cancer cells^42^. Their results revealed that SrTPO had no toxicity effects on Normal– NIH 3T3 cells in the opposite of doxorubicin. Delphi et al. demonstrated the anticancer effects of pectic acids on MDA-MB-231 breast cancer cells with inducing apoptosis and cell cycle arrest without significant effects on HUVEC non-cancerous cells^16^. Their results showed approximately 20 and 80% cell viability in MDA-MB-231 and HUVEC Cells after 24 h in 5 mg/mL of pectic acids, respectively. In agreement with our study, Li et al. demonstrated the percentage of apoptotic cells induced by oligosaccharides of apple was significantly more than polysaccharides in the dose and time-dependent manner^43^.

Our results revealed 10 and 80% cytotoxicity potential of 10 mg/mL pectin and unsaturated pectic oligosaccharides during 24 h, respectively. It is confirmed that some changes in the size and structure of natural polysaccharides can improve their bioactive properties. The results showed 32 and 92% cytotoxicity of 6 and 20 mg/mL POS, respectively.

Prebiotic and antibacterial effects of POS were also investigated in previous researches. Li et al. demonstrated the size-dependent prebiotic activity of POS on *Bifidobacterium infantis*, *Lactobacillus acidophilus* and its antibacterial effects *on Clostridium perfringens* and *Bacteroides fragilis*^44^. The antibacterial effects of POS were also revealed against *Staphylococcus aureus*, *Bacillus subtilis*, and *Escherichia coli*. According to the obtained results, prebiotic and antibacterial effects of POS were significantly higher than other prebiotics such as Fructo-oligosaccharides (FOS). Pectin and its derivatives as bio-resources for the production of various products is a new approach for commercial use of fruit waste.

## Conclusions

In this study, *Streptomyces hydrogenans* YAM1 was used for the degradation of pectin to unsaturated pectic oligo-galacturonic acids with more antioxidants and anticancer properties than pectin. The results demonstrated the potential benefits of *S. hydrogenans* YAM1 to make bioactive compounds from pectin degradation on one hand and removing fruit wastes from the environment on the other hand.

## Methods

### Isolation and identification of pectin degrading bacteria

The Cow fecal sample was used as an isolation source. It was cultivated in ISP2 medium containing yeast extract (4 g), malt extract (10 g), dextrose (4 g), agar (2 g), and 2.5 mL of 1% clotrimazole. The isolated *Streptomyces* strains were inoculated in 0.4 g K_2_HPO_4_, 0.008 g MgSO_4_, 0.2 g (NH_4_)_2_SO_4_, 0.008 g FeCl_3_, 0.1 g yeast extract, and 0.5 g pectin (101,845,988 Sigma) in 100 mL distilled water and incubated at 30 °C for 24 h. I/KI indicator was used to select the pectin-degrading colonies following the formation of a clear halo around the colonies^45^. The isolated strain was identified by the Iranian Biological Resource Center (IBRC). The universal primer pairs, 27F (5′-AGAGTTTGATCCTGGCTCAG-3′) as a forward primer and 1492R (5′-TACGGTTACCTTGTTACGACTT-3′) as a reverse primer, were used for amplification of 16S rRNA gene and then the sequencing was performed by Cosmogenetech, Korea. The sequence was submitted to NCBI, and the neighbor-joining method in MEGA X software was used to make the evolutionary history. The 16S rDNA sequence of *Lactococcus garvieae* was used as an outgroup (KF849269).

### Pectinase activity assay

Pectinase activity was determined at 40^˚^C, using the modified method of Bailey et al.^46^. A 0.5 mL of enzyme solution was incubated in the presence of 0.5 mL of 0.1 M apple pectin in 0.1 M acetate buffer pH 6, for 10 min. Then, according to Miller method, 1 mL of dinitrosalicylate (DNS) reagent was added to the solution, and the reaction tubes were boiled for 10 min, the absorbance values were read at 520 nm^47^.

### Analysis of obtained oligo-galacturonic acids

#### Thin layer chromatography

Thin layer chromatography (TLC) was used to detect the obtained products of pectin degradation. A 1.5 µL of the culture supernatants, and 1 mM solution of mono-galacturonic acids as a standard solution (purchased from Sigma) were spotted on silica gel 60 F254 (Merck). Chromatography was performed twice in n-butanol/acetic acid/water (2:1:2) as a mobile phase. For visualization, the dried spots on silica gel were sprayed with orcinol/sulfuric acid reagent (8 mg orcinol in 10 mL of 70% sulfuric acid). The plates were heated at 100 °C for 10 min^32^.

Matrix-assisted laser desorption ionization time-of-flight mass spectrometry (MALDI-TOF MS):

The oligosaccharides were analyzed by MALDI-TOF MS (Bruker Daltonics Microflex LRF). The samples (1 mL) were lyophilized and dissolved in water (100 μL). The soluble fraction of the samples were mixed with a matrix solution. The matrix solution was prepared by dissolving 50 mg of 2,5-dihydroxybenzoic acid (Sigma Aldrich) in 1 mL of 70% acetonitrile solution. The spectrum for each sample was obtained by collecting 100 shots per spot at five spots. Oligosaccharides were identified based on their theoretical m/z values with the addition of proton, Na^+^, K^+^, and NH_4_^+^^48^.

#### Detection of antioxidative effects of the oligo-saccharides

For analysis of the antioxidative effects of the oligo-saccharides, 0.5 mL of POS and pectin at various concentrations (0.6–20 mg/mL) was added to 2 mL of 0.2 mM methanolic solutions of DPPH (2,2-diphenyl-1-picrylhydrazyl). Henceforth, the reactions were stood for 30 min at 25 ˚C in dark condition. The absorbance of the samples was measured at 517 nm. The 0.2 mM DPPH solution was used as a control and the 60% ethanol was used as blank. Radical scavenging activity was calculated as following: % inhibition = [(Abs control –Abs sample)]/(Abs control)] × 100^38^. The tests were performed in triplicate.

#### Determination of the anticancer effects of POS on MCF-7 breast cancer cells

Human breast cancer MCF-7 cells (from the University of Isfahan Cell Line Bank) were cultivated in Dulbecco’s modified Eagle’s minimum media (DMEM; BioIdea), with 10% fetal bovine serum (BioIdea) and 1% penicillin–streptomycin solution (Sigma, USA). The cells were harvested onto six-well plates at a density of 1 × 10^4^ cells per well and incubated at 37˚C in 5% CO_2_ and 95% humidity for 24 h before the experiment^49^. The cells were then washed with phosphate-buffered saline (PBS, pH 7), and treated with fresh media containing 20 mg/mL of pectin, POS, and mono-galacturonic acid for 24. MTT ((3-(4,5-dimethylthiazol-2-yl)-2,5-diphenyl tetrazolium bromide) method was used to analyze the cell viability according to the Mosmann method^50^. To determine the viability percentages in POS treated cancer cells, the cells were harvested again in the fresh medium and treated with 6 and 20 mg/mL of POS. Henceforth, propidium iodide (PI, Sigma) was used for the detection of the immortalized cells. The cells were analyzed using Flow Cytometer (Becton Dickinson FACS Calibure) and the distribution of cells was determined using CellQuest software^51^.
